# Multiple myeloma: imaging evaluation of skeletal disease

**DOI:** 10.3402/jchimp.v3i2.21419

**Published:** 2013-07-05

**Authors:** Carlton Sexton, Candice Crichlow

**Affiliations:** 1 Department of Radiology, Medstar Union Memorial Hospital, Baltimore, MD, USA; 2 Department of Medicine, Medstar Union Memorial Hospital, Baltimore, MD, USA

**Keywords:** multiple myeloma, skeletal, pathological fracture, imaging

## Abstract

This patient is a 56-year-old woman with a history of IGG κ multiple myeloma diagnosed 15 years prior to admission. She had widespread lytic bone lesions and pathological fractures, which remarkably had not been accompanied by significant pain, but were mostly refactory to chemotherapy.

This patient is a 56-year-old African American female who still works as an elementary school principal with a history of IGG κ multiple myeloma diagnosed 15 years prior to admission, refractory to chemotherapy treatment. Previously, in 1992, she was diagnosed with monoclonal gammopathy of undetermined significance (MGUS) but was then diagnosed with multiple myeloma in 1997 when she was found to have a lytic lesion and compression fracture of the T7 vertebra. Subsequent treatment included radiation, dexamethasone, thalidomide, bortezimide, lenalidomide and bendamustine between 1997 and 2012. She was then treated with carfilzomib. Over the years, she developed chronic kidney disease due to Bence Jones proteinuria, anorexia, chronic anemia (requiring multiple blood transfusions), multiple electrolyte abnormalities and herpes zoster for which she remained on prophylaxis. She had widespread lytic bone lesions and pathological fractures, which remarkably had not been accompanied by significant pain.

Patients with myeloma commonly present with malaise and bone pain, and not uncommonly with a fracture that may result from a neoplastic mass or may result from osteoporosis induced by the plasma cell effect on bone marrow. Myeloma plasma cell infiltration results in bone resorption and osteolysis. The balance of bone osteoblastic and osteoclastic activity is upset. Osteoclast-activating factors secreted by the plasma cells begin a vicious cycle of osteolysis and tumor growth resulting from released cytokines. Imaging studies, old and new, are employed to diagnose the cause of bone pain, to help establish staging in the Durie–Salmon System, and to follow treatment. Radiographic skeletal surveys, computed tomography (CT), nuclear medicine, and MRI are all useful in an evaluation. Newer modalities, such as positron emission tomography (PET), have been used to study relapse.

Our patient suffers from one of four patterns of bone involvement, myelomatosis or diffuse skeletal involvement. Other forms are solitary plasmacytoma, diffuse skeletal osteopenia, and sclerosing myeloma. *Radiographic skeletal survey*, though insensitive for early disease, is positive in 75% of patients by the time a diagnosis is established. The axial skeleton, the spine, skull, pelvis, and ribs are more often affected than the limbs (Figs. 1–3). Lesions are almost always lytic. More than two clearly defined lytic lesions place patients like ours in Durie–Salmon Stage III disease. Therefore, the skeletal survey is recommended in all cases of newly diagnosed myeloma.

**Fig. 1:**
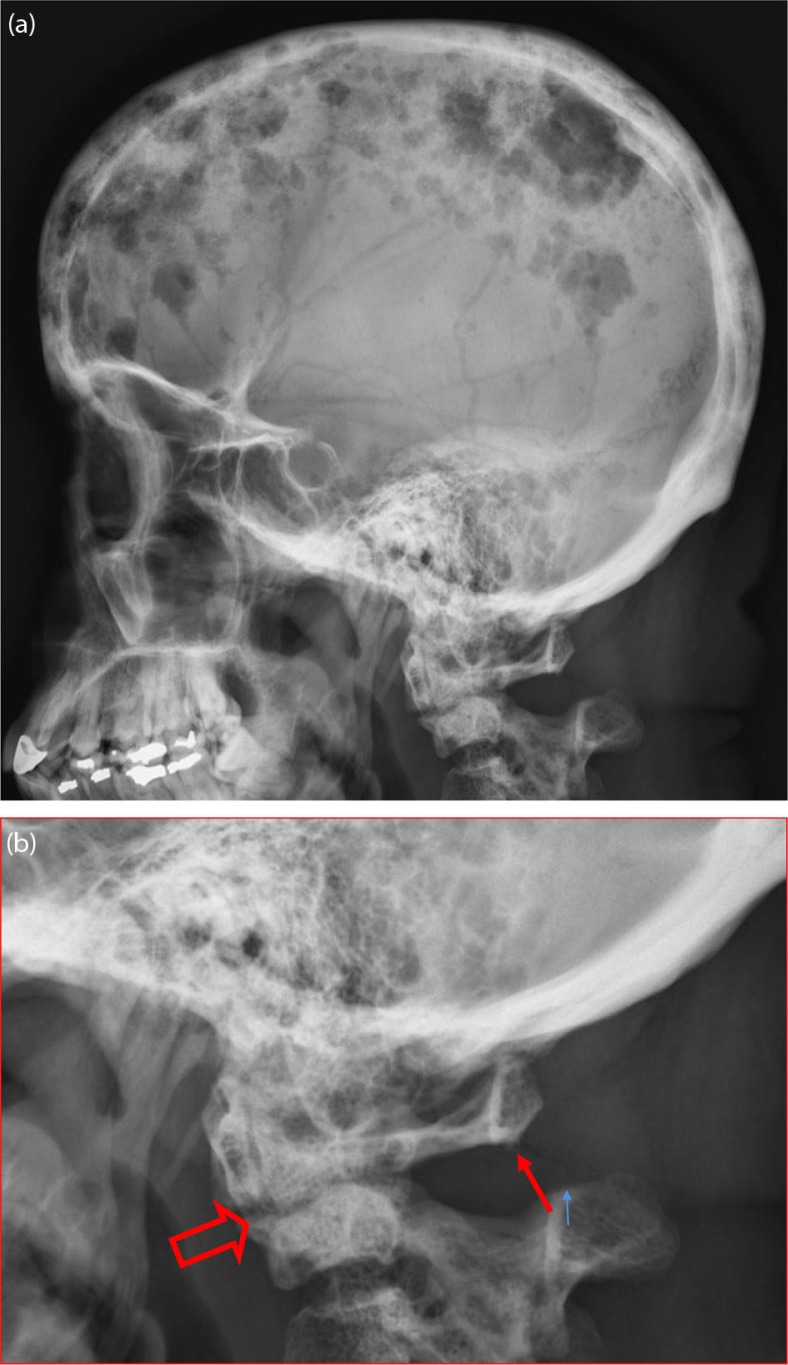
A lateral skull radiograph (a) shows numerous lytic lesions classic for myeloma. More subtle, at the craniocervical junction (b), there is a destructive lesion of C2 resulting in compression (open arrow), abnormal flexion (small arrow), and translation, and potential instability.

**Fig. 2:**
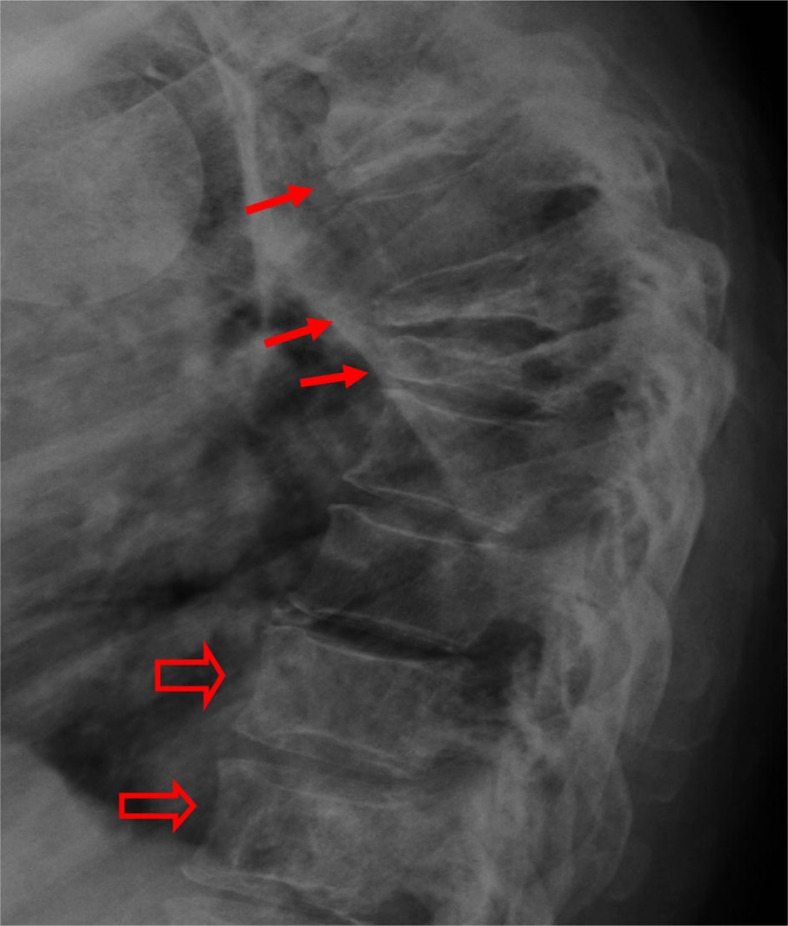
A lateral thoracic spine radiograph shows multiple severe vertebral body compression fractures (small arrows), vertebra plana, also characteristic of multiple myeloma. At T11 and T12 (open arrows), mild vertebral sclerosis is a result of treatment and healing.

**Fig. 3:**
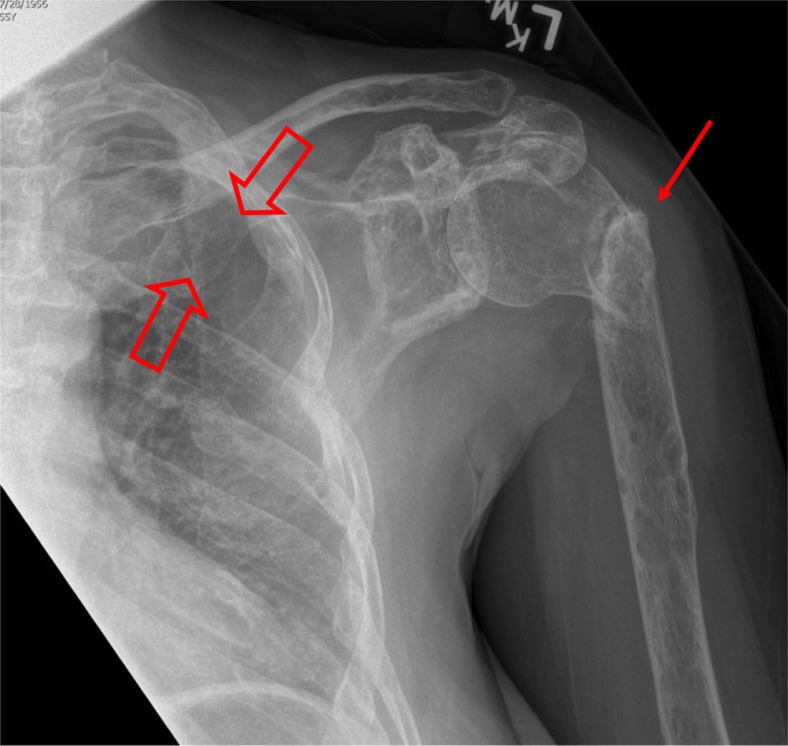
A frontal view of the shoulder shows a chronic pathological fracture of the surgical neck of the humerus (small arrow) across a large lytic lesion with a ‘moth-eaten’ appearance down the humeral shaft, and involving the clavicle and ribs. Note the missing 5th rib, destroyed by a plasmacytoma (open arrows).

*Computed tomography* (CT) has a role in clarifying ambiguous lesions, in assessing fracture risk by demonstrating the amount of residual cortical bone, and in guiding biopsy when needed. *Magnetic resonance imaging* (MRI) is used to establish the burden of disease, especially in the spine, that is prone to myeloma, and therefore at risk of fracture and spinal cord compression. In Stage III disease, patients with normal spine marrow pattern respond better to therapy and achieve remission more often. MRI is best and CT is second best in assessing the presence, the level, and the severity of myeloma-related spinal cord compression. The two techniques may be complementary in clarifying ambiguous lesions. Both MRI and CT detect extra osseous extension of disease.

Standard *radionuclide bone scan* with technetium 99-m relies on osteoblastic activity for tracer uptake, and myeloma is nearly exclusively osteoclastic. As many as half of myeloma lesions visible on radiographs are normal on the bone scan.

Positron emission tomography (PET) has a role in assessing the extent of extramedullary disease, and follow-up of these patients, but not in routine follow-up of treated myeloma in bone.

Spine fractures may be treated with *vertebroplasty/kyphoplasty*, and helpful in treating pain and spinal deformity. MRI is important to evaluate for potential pre-procedure spinal cord compression by a mass.

Multiple myeloma is becoming a more chronic disease, with life extended by successful chemotherapy. Knowing when, where, and why to use the full range of imaging described above will allow for accurate staging of the disease and to modify therapy as necessary, and to decrease the morbidity associated with fractures and spinal cord compression.
